# High altitude, hyper-arid soils of the Central-Andes harbor mega-diverse communities of actinobacteria

**DOI:** 10.1007/s00792-017-0976-5

**Published:** 2017-11-03

**Authors:** Alan T. Bull, Hamidah Idris, Roy Sanderson, Juan Asenjo, Barbara Andrews, Michael Goodfellow

**Affiliations:** 1 School of Biosciences, University of Kent, Canterbury, Kent, CT2 7NJ UK; 2 School of Biology, Ridley Building, Newcastle University, Newcastle upon Tyne, NE1 7RU UK; 3 Department of Chemical Engineering and Biotechnology, Centre for Biotechnology and Bioengineering (CeBiB), University of Chile, Beauchef 851, Santiago, Chile

**Keywords:** High altitude, Hyper-arid soils, Actinobacteria, β-Diversity, Microbial dark matter

## Abstract

The data reported in this paper are among the first relating to the microbiology of hyper-arid, very high altitude deserts and they provide base line information on the structure of actinobacterial communities. The high mountain Cerro Chajnantor landscape of the Central Andes in northern Chile is exposed to the world’s most intense levels of solar radiation and its impoverished soils are severely desiccated. The purpose of this research was to define the actinobacterial community structures in soils at altitudes ranging from 3000 to 5000 m above sea level. Pyrosequencing surveys have revealed an extraordinary degree of microbial dark matter at these elevations that includes novel candidate actinobacterial classes, orders and families. Ultraviolet-B irradiance and a range of edaphic factors were found to be highly significant in determining community compositions at family and genus levels of diversity.

**Electronic supplementary material:**

The online version of this article (10.1007/s00792-017-0976-5) contains supplementary material, which is available to authorized users.

## Introduction

The attraction of extreme environments for microbiologists has risen appreciably in recent years following recognition that the environmental limits to life on Earth are defined by the presence of microorganisms, the term environmental limits describing the outermost boundaries of the known physico-chemical world (Horikoshi et al. 2011). Reasons for studying the extremobiosphere (Bull 2011) in this context are several and encompass fundamental science and the hypothesis that they are likely to contain novel microorganisms that express new chemistry and metabolisms hence driving innovative biotechnology.

The most intensely researched habitats in the extremobiosphere have been hyper-thermal, deep sea and cryosphere and only more recently has attention turned to those exposed to long term and intense aridity that characterize deserts. Non-polar deserts constitute about one-third of the Earth’s landmass and our own research has been based in one of the world’s two coastal deserts, namely the Atacama Desert of northern Chile (Bull and Asenjo 2013; Bull et al. 2016). This desert is acknowledged to be the most arid on Earth and its aridity is maintained by a ‘rain shadow’ effect resulting from its enclosure between the Andean Cordillera and the Coastal Mountains. Hyper-arid and extreme hyper-arid environments (sensu Houston 2006) are characteristic of the desert’s core region but the Atacama also comprises salars, Altiplano and high altitude mountain plateaus, and volcanoes.

Reports of the microbiology of high altitude mountain soils worldwide are sparse and little information is available relating to those of the Central-Andes. However, several investigations of high altitude wetlands (ponds, saline lakes, salterns and peatlands) at altitudes between ca. 3700 and 6500 m above sea level have been made in the Chilean and Bolivian Altiplano and the Central-Andes of Argentina. One such initiative, the multidisciplinary High-Lakes Project, has as a primary objective the assessment of increased environmental stress on the biological disturbance of high altitude lakes during the periods of rapid climate change as an analogy to early Mars (Cabrol et al. 2009). Other studies have started to chart the diversity and novelty of particular microbial groups (archaea and bacteria, Dorador et al. 2010, 2013; Aszalós et al. 2016; diatoms, Blanco et al. 2013), to explore polyextremophily (Albarracin et al. 2015) and diurnal community dynamics in response to solar radiation (Molina et al. 2016) in these elevated wetland habitats.

The most intensive investigations of barren high altitude soils per se have been made by Steve Schmidt and his group, several of which have been sited on the highest volcanoes in the Atacama region (Lynch et al. 2012) which they consider to be “arguably the most extreme soil ecosytems on Earth” based on extreme temperature cycling, aridity, low atmospheric pressure and extreme solar radiation. In a series of papers Schmidt and his colleagues reported pioneering studies of the microbiota of fumarolic and non-fumarolic soils of the Socompa and Llullaillaco volcanoes between 5500 and 6330 m a.s.l. These communities are among the simplest found in terrestrial ecosystems and appear to be sustained by the activities of facultative chemoautotrophic bacteria (Costello et al. 2009; Lynch et al. 2012).

This paper reports the latest results of our on-going research on desert soil actinobacteria, the research landscape being the Chilean high mountain desert, Cerro Chajnantor (5640 m a.s.l.), which lies east of San Pedro de Atacama. This landscape is subject to a combination of extreme environmental conditions including the world’s highest levels of surface UV radiation (Cordero et al. 2014). In this study metagenomic profiles of actinobacterial communities in these very high altitude desert soils were compared with those of low altitude desert soils, previously defined by (Idris et al. 2017a), to test the hypothesis of microbiome taxonomic distinctiveness of Atacama Desert soils. In addition, attempts have been made to identify the principal determinants of actinobacterial community structure as a function of soil environment and climate characteristics.

## Materials and methods

### Research landscape and sample collection

**Fig. 1:**
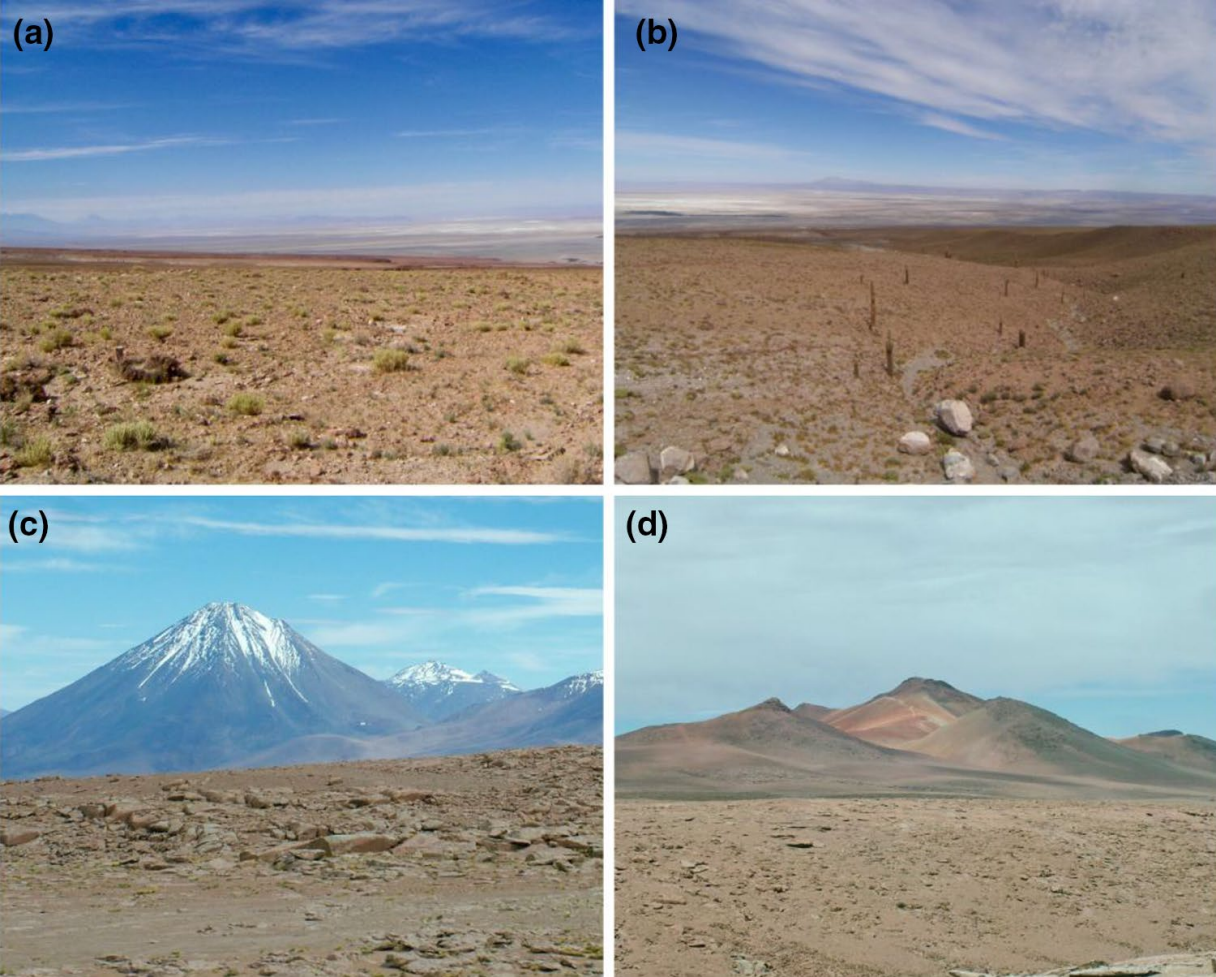
Sampling locations on the Cerro Chajnantor. **a** ALMA 1 site at 3018 m a.s.l. with Salar de Atacama in the background. **b** ALMA 3 site at 4000 m a.s.l. showing scattered colonization by *Echinopsis atacamensis.* **c** Landscape between ALMA sites 3 and 5 with Licancabur Volcano in the background. **d** ALMA 5 site at 5041 m a.s.l. on the Chajnantor Plateau with the peak of Cerro Chajnantor in the background

Replicate samples (4) were collected aseptically in October 2012 and stored as described previously (Idris et al. 2017a). Surface and subsurface samples were taken at three altitudes on the Cerro Chajnantor (Table 1). At the two lower altitudes the ecosystems contained sparse vegetation largely comprising Andean grasses, xerophytes and columnar cacti, particularly *Echinopsis atacamensis* (Fig. 1). The highest sampling site (5041 m a.s.l.) lay within the periglacial zone as defined by Schmidt et al. (2012).

**Table 1:** Cerro Chajnantor research site data

Location/sampling depth (cm)	Latitude °S	Longitude °W	Altitude (m a.s.l.)	Atmospheric pressure (bar)	Air temp (°C)	Radiation (kJ/m^2/^day)		Vegetation
						UV-A^a^	UV-B^b^	
ALMA 1 (2)	23°04′39″	67°57′43″	3018	1015	25.0	1545	46.0	Present
ALMA 2 (30)	23°04′39″	67°57′43″	3018	1015	25.0			
ALMA 3 (2)	23°03′31″	67°52′27″	4000	1015	27.2	1579	49.0	Present
ALMA 4 (30)	23°03′31″	67°52′27″	4000	1015	27.2			
ALMA 5 (2)	23°00′49″	67°45′31″	5041	1015	15.6	1621	51.2	Absent
ALMA 6 (30)	23°00′49″	67°45′31″	5041	1015	15.6			

^a^315–400 nm

^b^280–315 nm

The Cerro Chajnantor is a lava dome and part of a large volcanic complex formed on top of a pyroclastic shield. It is one of several volcanic peaks that border the Chajnantor Plateau (ca. 5000 m a.s.l.), a region of the southern Altiplano that is the location of a group of astronomical observatories including the Atacama Large Millimeter Array (ALMA). ALMA is operated as an international partnership of which the European Southern Observatory (ESO) is a member. Permission to collect samples was given by the ESO and in accordance with its requirements health checks were made at the ALMA Operations Support Facility (2900 m a.s.l.) prior to sampling at higher altitudes.

### Soil analyses and climate data

Soil measurements and elemental analyses were made at the Geochemistry Laboratory, University of Chile, Santiago; and UV irradiance data were collected at the Chajnantor Plateau in February–March 2015 by Dr. Raul Cordero (Department of Physics, University of Chile, Santiago). Latitude, longitude, elevation and other climatic data were collected during sampling using a handheld multi-function altimeter (AltiTech2, Highgear Inc., Durham, NC, USA).

## DNA extraction, PCR amplification and pyrosequencing

All of the procedures used were those described by us previously (Idris et al. 2017a).

### Bioinformatic and multivariate statistical analyses

A total of 12,388 valid pyrosequence reads were processed using CLCommunity v3.30 software at Chunlab Inc. (http://www.chunlab.com; Seoul National University, Korea) and taxonomic assignments to actinobacterial family and genus ranks were made using the EzBioCloud (Yoon et al. 2017) database (see Idris et al. 2017a for details of these procedures).

Alpha-diversity analyses including rarefaction, rank-abundance, Shannon diversities and Chao1 richness estimations were calculated using CLCommunity software. Operational Taxonomic Unit tables (OTU) were saved in comma delimited (.csv) format prior to further analysis. Identified generic phylotypes were checked manually from OTU tables and analyzed to determine their composition in each of the sample sites. Venn diagrams were constructed using the Mothur version 1.37.4 program (http://www.mothur.org/; Schloss et al. 2009). Beta-diversity analyses of actinobacterial communities were made using unconstrained principal components (PCA) and constrained redundancy (RDA) analyses using the “vegan” (Oksanen et al. 2013) package to explore the effects of environmental variables on community composition. The ANOVA permutation test (Ter Braak 1986) was used to assess the significance of such variables on community structure.

## Results

### Site characteristics

The texture of sampled material varied from coarse soil, to gravel, regolith, and rock fragments the moisture contents of which were zero with the exception of the subsurface at the highest altitude (Table 2), the latter reflecting its periglacial nature. Ultraviolet irradiance levels are quoted as doses (kJ/m^2^) and computed by integrating through time to provide a daily dosage. UV-A and UV-B irradiance increased by 4.9 and 11.3% over the altitude range sampled (Table 1), values agreeing closely with those reported in a major survey of solar spectral conditions in the Atacama Desert, including the Chajnantor Plateau (Cordero et al. 2016). On the basis of redox potential values (Husson 2013) all of the soils could be designated as dry and moderately oxidizing and showed little inter-site variation. The nature of possible oxidants in these soils has not been investigated but in part may be related to very high UV radiation flux. Soil conductivity has a strong correlation with salinity and also correlates with soil texture and particle size, an effect that was particularly noticeable in the very low values recorded at the 5041 m sites. All of the soils contained relatively high concentrations of Ca and Na that are indicative of high salt contents, a condition of Atacama Desert soils regarded as indicative of persistent aridity (Crits-Christoph et al. 2013) (Table 2).

**Table 2:** Physico-chemical characteristics of Cerro Chajnantor soils

Location	pH	Redox potential (mV)	Conductivity (μS/cm)	Moisture (%)	Total organic content (%)	Texture	Elements (%)				
							Ca	Mg	Na	K	Fe
ALMA 1	7.08	360	215	0	1.67	Coarse mineral soil	3.9	1.3	2.7	1.8	4.8
ALMA 2	7.63	336	326	0	3.67	Coarse mineral soil	3.2	1.3	2.0	2.2	3.9
ALMA 3	6.55	360	239	0	3.33	Regolith	3.2	1.3	2.6	1.9	4.5
ALMA 4	6.70	335	108	0	3.67	Gravel	3.0	1.5	2.4	1.8	4.5
ALMA 5	6.73	369	54	0	2.00	Regolith/rock	3.6	1.4	2.4	2.7	4.1
ALMA 6	6.30	365	44	4.67	0.67	Gravel	3.5	1.4	2.4	2.7	4.1

### Community coverage and composition

Our pyrosequencing programme produced an average of 2956 sequence reads per soil sample most of which had reads of > 1000; this average read number is smaller than that we obtained for low altitude Atacama soils using identical procedures (Idris et al. 2017a) but compares very favorably with low altitude Atacama soil surveys reported earlier by Crits-Christoph et al. (2013).

**Fig. 2:**
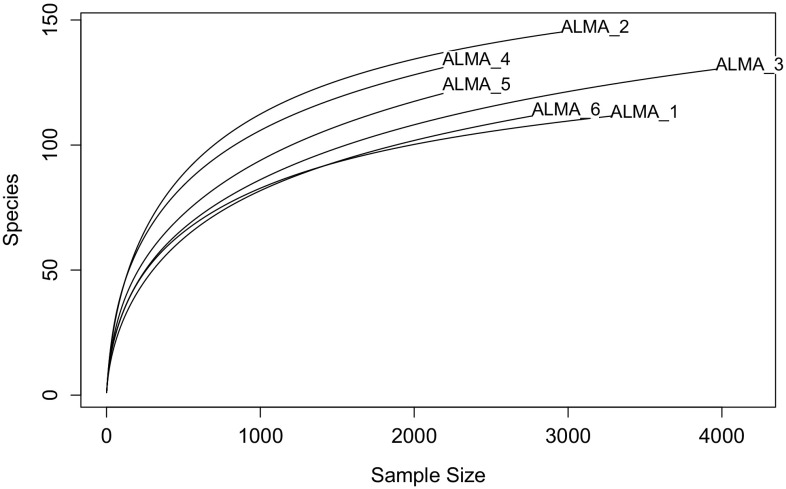
Rarefaction analyses of ALMA OTUs at 94.5–97.0% phylogenetic similarity showing the relation between the increase in generic richness and the number of randomly sampled sequences

Inspection of rarefaction curves (Fig. 2) shows that they tend to asymptotes for each of the soils thereby arguing for high sequence coverage, a conclusion strongly supported by reference to Good’s coverage metric (Table 3). With the exception of the subsurface gravel from 3018 m a.s.l. (ALMA 2), the Shannon and Simpson Indices do not provide any evidence for dramatic changes in generic diversity as a function of altitude, although generic richness in terms of observed OTUs and Chao estimates showed greater variability (Table 3). The reciprocal of Simpson’s index is sensitive to the level of dominance in microbial communities but only in the 3018 m surface and the 5041 m subsurface soils were dominant profiles evident (1/*D* < 50; Zhou et al. 2002) while the remainder possessed uniform community profiles.

**Table 3:** Observed generic OTUs and α-diversity indices

Location	Good’s library coverage (%)	Observed OTUs	Chao 1	Shannon index (*H*)	Simpson diversity (1/*D*)
ALMA 1	97	158	195	4.03	33.3
ALMA 2	95	354	484	5.15	100
ALMA 3	98	211	256	4.41	50
ALMA 4	95	233	373	4.59	50
ALMA 5	95	224	331	4.52	50
ALMA 6	97	207	257	4.12	33.3

When sequences were assigned to OTUs, 55 families and 234 genera of actinobacteria were defined; of these 33 and 41%, respectively, could not be assigned to valid named taxa and hence are considered to be putatively novel candidate families and genera (Supplementary Material, Tables 1 and 2). A significant proportion (20%) of identified families could be assigned to deep lineage actinobacteria belonging to the class *Acidimicrobia* (Norris 2012), a majority of which appear to be novel. As in our previous report (Idris et al. 2017a) we have used a rank-abundance threshold of 0.1% to define a rare actinobacteria biosphere (sensu Lynch and Neufeld 2015); using this criterion 40% of detected families and 65% of detected genera belong to the rare microbiosphere.

### Taxonomic diversity and variation between sites and depths

The extraordinary novelty among high taxonomic rank actinobacteria occurring in low altitude Atacama Desert ecosystems (Idris et al. 2017a) was found to be comparable at the Cerro Chajnantor sites, with phylotypes representing 2 putatively new classes, 4 new orders and 18 new families in the phylum *Actinobacteria* being recorded (cumulative site data, Supplementary Material, Table 1). The large majority of OTUs were identified as members of the orders *Frankiales* (34%), *Micrococcales* (9%), *Acidimicrobiales* (7%), *Propionibacteriales* (7%) and *Pseudonocardiales* (5%); sequence reads corresponding to OTUs of a putative new order FJ478799_o accounted for 2.5% of the Chajnantor community.

**Fig. 3:**
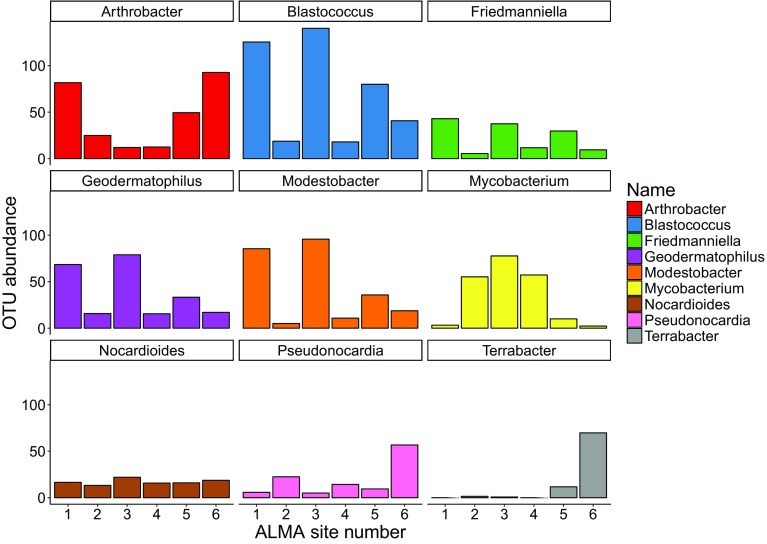
Effects of altitude and soil depth on the abundance of the most dominant genera; sites 1 and 2 at 3018 m a.s.l.; sites 3 and 4 at 4000 m a.s.l.; sites 5 and 6 at 5041 m a.s.l.. Samples at sites 1, 3, 5 taken at 2 cm depth; sites 2, 4 and 6 at 30 cm depth

The most frequently detected genera comprizing 64% of the actinobacterial phylotypes were in order of abundance: HQ910322_g, *Blastococcus*, *Arthrobacter*, *Modestobacter*, *Geodermatophilus*, *Mycobacterium*, HQ674860_g, JF266448_g, *Friedmanniella*, FJ479147_g, *Pseudonocardia*, *Nocardioides*, *Jatrophihabitans, Terrabacter* and *Sporichthya* (Supplementary Material, Table 2). The abundance of certain genera (*Blastococcus*, *Modestobacter*, *Geodermatophilus*, *Friedmanniella*) was clearly related to soil depth and to altitude (Fig. 3) and probably reflects an ability to withstand irradiation stress; in all cases abundance fell progressively with altitude. All of these organisms are contained in the Terrabacteria group of prokaryotes (Battistuzzi and Hedges 2008) that is distinctive of hyper-arid deserts and notable for containing organisms selected to withstand environmental stress including irradiations and desiccation. Gtari et al. (2012) studied stress phenomena in selected strains of these actinobacterial clades and found high tolerance to UV radiation especially in melanized *G*. o*bscurus* and *M*. *multiseptatus* but less so in *B*. *saxobsidens* that produces orange pigmentation. Protective UV-absorbing mycosporine-like amino acids have recently been reported in a member of the genus *Pseudonocardia* (Miyamoto et al. 2014), another of the most abundant actinobacterial taxa in Chajnantor soils. Also of note is that most of the identified high abundance generic phylotypes are known to produce coccoid cells and in some cases spores, morphological features that Gtari et al. (2012) opine may reflect adaptations to arid habitats.

The proportion of identified co-occurring generic phylotypes in surface and subsurface soils at each altitude was approximately 50%; however, greater differentiation of actinobacterial communities was evident in between-surface and between-subsurface communities where co-occurrence was only 30% (Supplementary Material, Figure 1). Sub-surface communities tended to be more taxonomically rich but the differences were small (< 10%). Previously unreported phylotypes were detected in all of the soils.

**Fig. 4:**
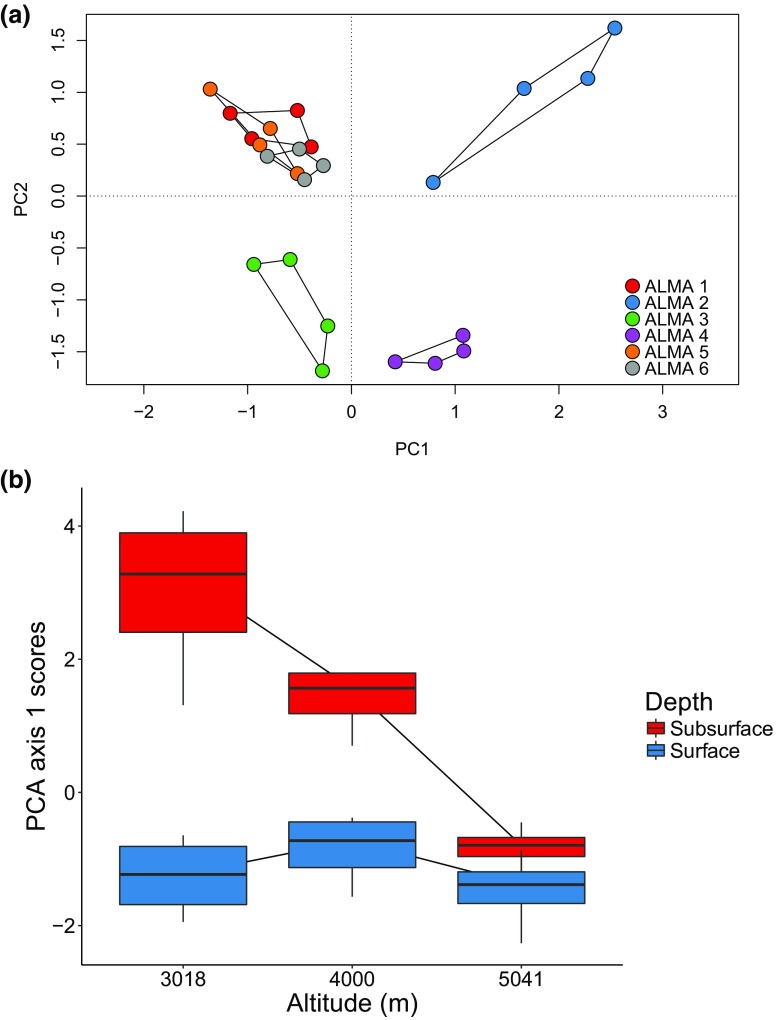
**a** Principal component analysis of surface and subsurface communities. PC1 and PC2 contributed 15 and 11% of the variance, respectively, **b** box plot summarising the status of actinobacterial communities with regard to altitude and sample depth. Interquartile ranges are represented by box outlines, median values by midlines, and maximum and minimum values by whiskers

The total variation explained by the first two principal component axes was relatively low at 26%, but nevertheless distinct patterns could be observed. Two broad community groups can be defined (Fig. 4a): Group 1 comprised those communities at ALMA sites 1, 5 and 6 (upper left of the PCA plot), while the more diffuse Group 2 comprised communities at ALMA sites 2, 3 and 4. Substantial within-site variation in community composition was evident at some of the sites (ALMA 2, subsurface, 3018 m, and ALMA 3, surface, 4000 m) as indicated by the larger spread of polygons in PCA ordination space (Fig. 4a); other sites had relatively little variation in composition between replicate samples. The PCA notably differentiates surface and subsurface communities at the lower elevations. The overall trends are clearly discerned in the box plot presentation of the data that summaries changes in PCA axis 1 scores with altitude and sampling depth (Fig. 4b); as altitude increased there was a gradual convergence in community composition between the surface and subsurface samples, as well as a decline in within-community variability of taxonomic composition.

At the lowest altitude (3018 m), there are major differences in the community composition depending on depth, and also more variability between samples in the subsurface (ALMA 2) than surface (ALMA 1). The surface samples, that are subject to high levels of UV radiation, did not appear to change much in community composition as altitude increased from 3018 to 5041 m, as their PC Axis 1 score remained constant, with a similar amount of variation around the mean. In contrast, the community composition of subsurface samples (ALMA 2, ALMA 4 and ALMA 6) changed dramatically with altitude, with the PC Axis 1 score gradually converging to that of the surface samples by 5041 m, indicating that at the highest altitudes surface and subsurface samples have similar actinobacterial community composition. Moreover, individual subsurface samples became less variable with increasing altitude suggestive of increased stresses at high altitude even within the subsurface environment.

### Beta-diversity

The two actinobacterial community groups suggested by principal components analysis were confirmed by the construction of a double annotated hierarchical heat map based upon the 138 validated generic phylotypes (Supplementary Material, Figure 2) and, moreover, the majority of the previously most frequently detected and abundant phylotypes (Supplementary Material, Table 1) were assignable to the Group 1 communities. The heat map Group 1 communities were dominated by members of the family *Geodermatophilaceae* and the genera *Friedmanniella, Sporichthya* and *Terrabacter*, while other phylotypes were co-dominant in the two community groups, notably the genera *Jatrophihabitans, Nocardioides* and *Pseudonocardia*. These dominant phylotypes were most conspicuous in surface soil sites of the Group 1 communities, i.e. at 3018 and 5041 m a.s.l., again suggesting an enhanced tolerance to radiation stresses.

**Fig. 5:**
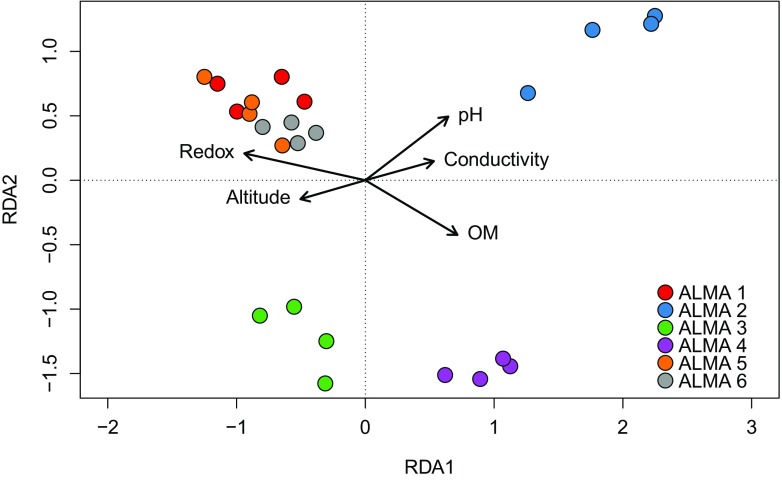
Redundancy analysis biplot showing relationships between identified actinobacterial communities and environmental variables. Surface samples are ALMA 1, 3 and 5; subsurface ALMA 2, 4 and 6. RDA1 explains 12% and RDA2 9% of the variation

Relationships between community composition and environmental variables were examined using redundancy analysis (RDA), the results of which are summarized in Fig. 5. In the RDA ordination diagram, the length of the arrow for each constraining variable indicates the magnitude of its effect, arrows pointing in the same direction are positively correlated with each other; arrows at 90° to each other are uncorrelated, and arrows at 180° are negatively correlated.

Samples positively associated with constraining variables are located near the arrow heads. Of the variables tested, soil redox potential and organic matter are the most important drivers of community composition (although they are negatively correlated with each other) and are positively correlated with altitude and soil conductivity, respectively. Soil pH appeared to be correlated only with the variable subsurface soil samples at 3018 m (see Fig. 5) which was the most alkaline of all those tested. Results of RDA permutation ANOVA tests showed decisively that all of the environmental variables were highly significant in determining actinobacterial community compositions in the Cerro Chajnantor landscape (Table 4). Large *F* ratios indicate significant results and *p* values < 0.05 refute the null hypothesis that community composition is unrelated to environmental variables.

**Table 4:** RDA permutation test of Cerro Chajnantor environmental variables

Environmental variable	*F* ratio	*p* value
Altitude	1.6716	0.008
UV-B dosage	2.1011	0.014
pH	3.0075	0.001
Redox	2.2539	0.001
Conductivity	1.6550	0.004
Organic matter	1.6143	0.018

*F* ratios based on 999 permutations

**Fig. 6:**
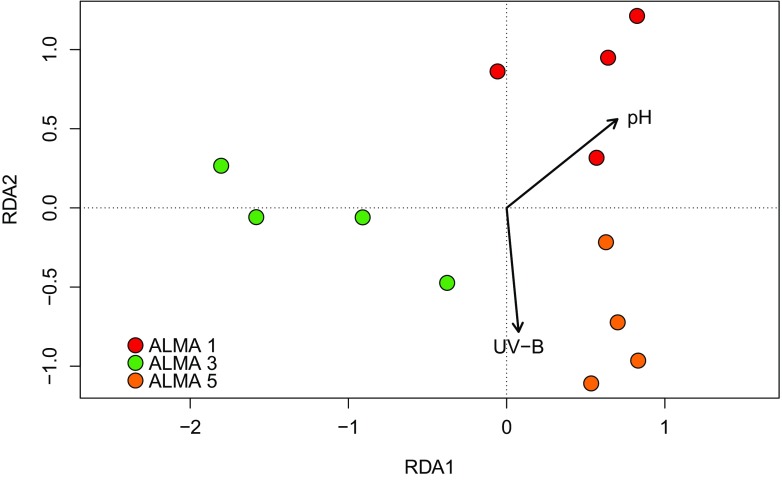
Redundancy analysis biplot showing relationships between identified actinobacterial communities and UV-B irradiance. ALMA 1 = 3018 m; ALMA 3 = 4000 m; ALMA 5 = 5041 m. RDA1 explains 23% and RDA2 9% of the variation

The strong positive correlation between redox potential and altitude and its association with the Group 1 communities was particularly noteworthy given the fact that Atacama Desert soils have long been known to possess strong oxidizing properties (Navarro-Gonzalez et al. 2003) although the nature of the oxidant(s) has remained elusive. Recently, however, Georgiou et al. (2015) have demonstrated that photo-generated metal superoxides and peroxides accumulate in top soils in the Yungay location of the Atacama Desert and opine that they furnish some key elements of reactive oxygen species in desert soils. Based on data contained in Cordero et al. (2016) we estimate that radiation levels imposed on Cerro Chajnantor soils to be approximately 17% greater than those at the Yungay sites. Subsequently we have used redundancy analysis to examine the relationship of community composition with UV-B irradiance (Fig. 6) and found it to be singularly correlated at the highest altitude.

When actinobacterial family distributions are superimposed on the RDA biplot (data not shown), those most strongly correlated with UV-B irradiation were found to be the *Corynebacteriaceae, Microbacteriaceae* and *Pseudonocardiaceae,* and to a lesser degree the *Nocardiaceae* and *Sporichthyaceae*. Perhaps surprisingly the *Geodermatophilaceae* whose phylotypes dominated the Group 1 communities were negatively correlated with UV irradiation. Reports on UV resistance of desert bacteria tend to be rather anecdotal and little information is available on resistance mechanisms per se. Early studies showed that UV resistant actinobacteria often dominated desert communities such as rock varnish (Kuhlman et al. 2005) and that *Geodermatophilus* isolates in particular were resistant to high radiation dosage (Rainey et al. 2005). The latter authors remarked that “The recovery of large numbers of extremely ionizing-radiation-resistant bacteria from an arid soil and not from a non-arid soil provides further ecological support for the hypothesis that the ionizing-radiation resistance phenotype is a consequence of the evolution of other DNA repair systems that protect cells against commonly encountered environmental stressors, such as desiccation”. Recently the UV-C resistance of bacteria isolated from the Atacama and Sonoran Deserts was found to be greater than or comparable to that of *Deinococcus radiodurans* (Paulino-Lima et al. 2016). Significantly all resistant isolates had intracellular manganese/iron ratios very much higher than those of sensitive ones although resistance was not correlated with the availability of Mn or soil Fe/Mn ratios. Despite the fact that the soils studied by Paulino-Lima et al. (2016) and those of the Cerro Chajnantor sites contain only low concentrations of Mn, mechanisms have been identified for its uptake and accumulation from low Mn environments (Sun et al. 2010). It remains to be investigated whether or not high altitude actinobacteria reported in this paper resist extreme UV stress by means of manganese transport and regulation systems to control recovery from radiation injury as proposed by Daly et al. (2004).

## Discussion

Previously we postulated the existence of a core actinobacterial microbiome in low altitude Atacama soils that may play a key role in ecosystem function (Idris et al. 2017a); the composition of this core microbiome accords very closely with that defined for the Cerro Chajnantor sites in which phylotypes of the following families were dominant (percentage dominance in brackets): *Geodermatophilaceae* (19.6), HQ910322_f (9.5), *Acidimicrobiaceae* (8.3), *Pseudonocardiaceae* (7.9), *Micrococcaceae* (5.9), *Nocardioidaceae* (5.8), *Microbacteriaceae* (5.5), and *Micromonosporaceae* (5.3). The one anomaly was Candidatus FJ479147_f that was the most abundant family (18.4% total phylotypes) in low altitude Atacama soils but had relatively low abundance in the Chajnantor (2.6%).

Very few microbiological studies have been made of nutrient poor, high altitude desert soils and of the biological and edaphic pressures that select for community structures. We have sought to reveal adaptive traits that are plausibly associated with the fitness of actinobacteria to high altitude Atacama Desert soils to promote what Lynch et al. (2014) have reasoned “the testing of hypotheses for future culture-based experiments”. These authors have pointed to the Gram-positive cell wall architecture and widespread capacity to sporulate as possible early adaptations to arid environments and it is notable that a high proportion (30%) of the most dominant actinobacterial phylotypes identified in Cerro Chajnantor soils are amycelial and/or known spore producers. Although UV-B accounts for only a small fraction of total solar irradiation, dosage levels on the Cerro Chajnantor are the world’s highest recorded and its effect is greatly enhanced as a consequence of extremely low values of precipitable water (< 1 mm), total ozone column and atmospheric aerosols, and high altitude (Cordero et al. 2014, 2016). Figure 6 pointedly indicates that the highest altitude surface soil (ALMA 5) correlated most strongly with UV-B dosage (near the tip of the UV arrow) whilst the ALMA 1 soil (lowest altitude) is at the top of the graph with lowest UV levels. Whereas our finding of a high abundance of *Geodermatophilaceae* phylotypes at this highest altitude site accords with the ability to isolate members of this family from other desert top soils and from exposed stone surfaces (Busarakam et al. 2016; Gtari et al. 2012; Urzì et al. 2001), redundancy analysis failed to establish a correlation with UV-B irradiance. It is feasible, therefore, that the abundance of these bacteria in Cerro Chajnantor soils may be correlated with their resistance to other stress factors that give rise to reactive oxygen species (ROS) such as severe desiccation and soil redox potentials, a proposition that is supported by the highly significant results of redundancy analyses (Table 4). The redundancy analysis reveals a large amount of unexplained variation in our data (over 70%). Some of this unexplained variation may be a result of unmeasured environmental variables: for example a more detailed assay of the chemical composition of the underlying geology at the six sampling sites may have provided additional explanatory variables to include in the RDA. Nevertheless, it is likely that actinobacteria in extreme desert environments are subject to considerable spatial and temporal environmental stochasticity (Caruso et al. 2011), adding to the variation in community composition.

A salient outcome of Schmidt’s metagenomic surveys of Atacama and other Andean volcanic soils was the hypothesis that organic carbon could be generated by chemoautotrophic microorganisms thereby sustaining communities in such harsh, nutrient impoverished environments. Samples from the Llullaillaco Volcano 6034 m a.s.l. were dominated by actinobacterial phylotypes among which a *Pseudonocardia* species was the most abundant (Lynch et al. 2014), the genome of which encoded for CO_2_ fixation via the Calvin cycle and complete pathways for C1 compounds, hydrogen, ammonia and nitrate assimilation. The family *Pseudonocardiaceae* is the fourth most abundant in Cerro Chajnantor soils and in 5041 m samples the abundance of a *Pseudonocardia* phylotype reached 13% (Supplementary Information, Table 2). The high incidence of *Arthrobacter* and *Terrabacter* phylotypes at this highest altitude site investigated (8 and 21% abundance, respectively; Fig. 3) is also notable given reports of carboxydovory in these genera (Kim et al. 2011; Lalonde and Constant 2016).

Future research priorities undoubtedly include the identity and characterization of adaptive traits in these high altitude desert communities. An immediate opportunity is afforded by the collection of isolates representing several of the dominant actinobacterial families, including the families *Geodermatophilaceae* and *Pseudonocardiaceae*, for culture-based physiology investigations and whole genome mining. To date we have whole genome sequences for two cultured members of the *Geodermatophilaceae* (*Modestobacter caceresii*, Busarakam et al., 2016, and *Geodermatophilus atchiseltius* sp. nov., J. F. Castro et al., unpublished data) both of which encode a high proportion of environmental stress related genes and others associated with chemolithotrophic metabolism. However, members of many of the most abundant lineages such as HQ910322_f have yet to be brought into culture or even characterized taxonomically and given the extent of the actinobacterial dark matter in the Cerro Chajnantor soils this is a major task. Future soil surveys based on a multi-omics approach in which metagenomics is complemented by metatranscriptomics and metaproteomics should enable active and dormant actinobacteria in the communities to be distinguished (Aanderud et al. 2015) and to provide insights into the functional genomics of habitat adaptation as demonstrated by Lynch et al. (2014).

## Conclusions

The Atacama Desert landscape is an extraordinary repository of actinobacterial ‘dark matter’ with significant numbers of Candidatus taxa detected at supra-generic levels and nearly 50% of phylotypes not being assigned to validly named genera. Similarly high are our estimates of the actinobacterial rare biosphere in these high altitude environments, measuring 65% of all detected generic phylotypes.

At supra-genus rank 2 putatively new classes, 4 new orders and 18 new families in the phylum *Actinobacteria* have been defined and such, we opine, represents mega-diversity.

The extremely low aerosol optical depth values were recorded at high altitude sites in the Atacama Desert, particularly on the Chajnantor Plateau, (Cordero et al. 2016) has implications for habitat contamination by deserts dusts blown in either from local or distant geographic sites and is strongly indicative that the microbial communities defined in this study can be categorized as indigenous.

The actinobacterial communities found in extreme arid habitats of the Atacama Desert present unique opportunities for bioprospecting. Thus, initial screening of the Cerro Chajnantor culture collection has shown that approximately 20% of all isolates gave positive results in a *Bacillus subtilis* reporter strain survey, while a new species of the rare genus *Lentzea*, *L. chajnantorensis* (Idris et al. 2017b) isolated from the 5041 m site, produces novel diene and monoene glycosides some of which have anti-HIV integrase activities (Wichner et al. 2017).

## Electronic supplementary material

Below is the link to the electronic supplementary material.

Supplementary material 1 Supplementary Fig. 1 Hierarchical heatmap showing actinobacterial distribution of validly published genera among the six ALMA soil samples. The double hierarchical dendrogram shows the actinobacterial distribution and the heatmap represents the relative percentage of each actinobacterial genus within each sample. The relative abundance values are indicated by the colour intensity as shown in the legend on the top left corner (JPEG 1435 kb)

Supplementary material 2 (XLSX 14 kb)

Supplementary material 3 (XLSX 24 kb)
