# Supplementary figures and images for: High altitude, hyper-arid soils of the Central-Andes harbor mega-diverse communities of actinobacteria
Source: Extremophiles. 2017 Nov 3;22(1):47–57. doi: 10.1007/s00792-017-0976-5 (PMC5770506; doi:10.1007/s00792-017-0976-5)

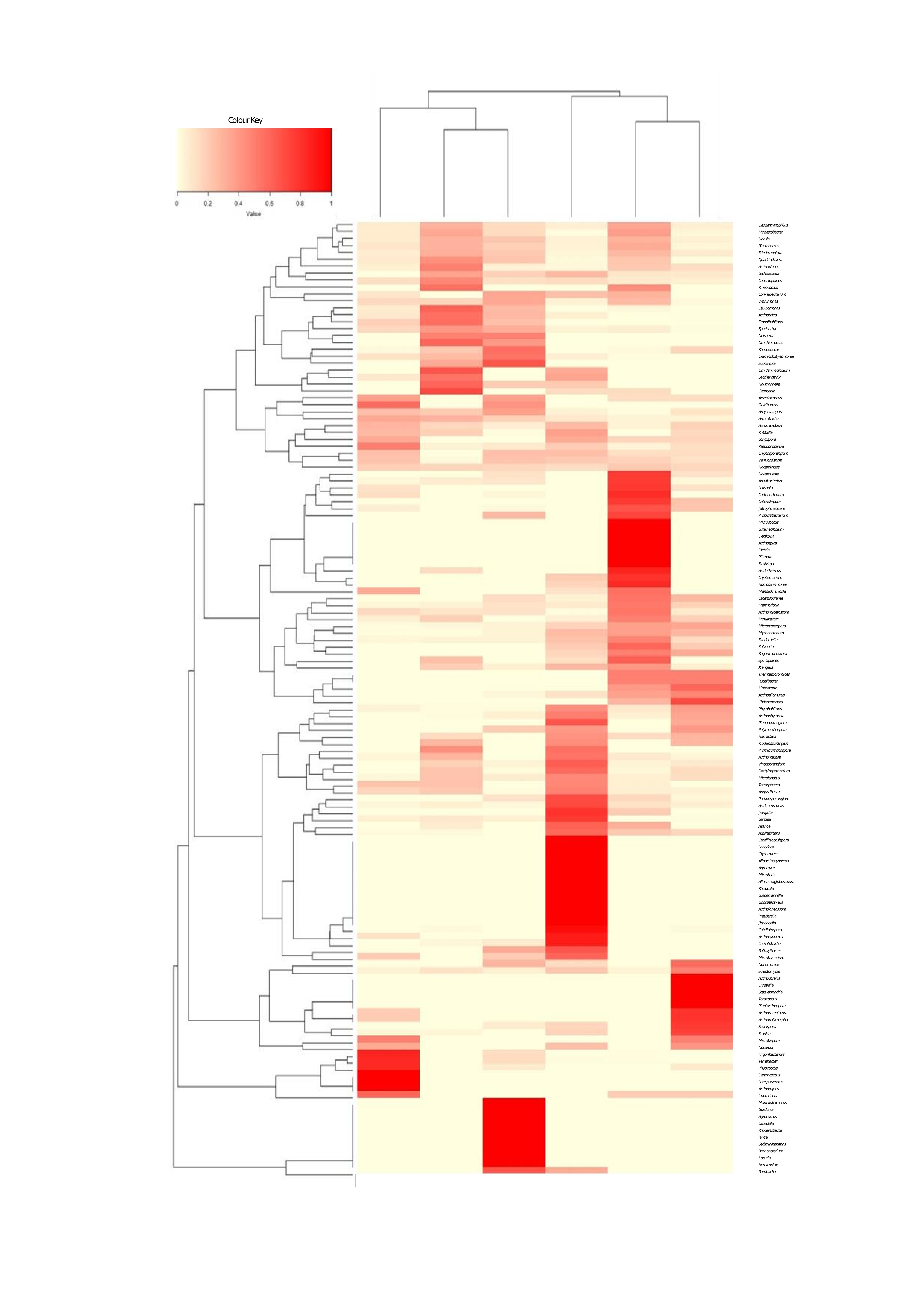

Supplement: Supplementary file 1 — Supplementary material 1 Supplementary Fig. 1 Hierarchical heatmap showing actinobacterial distribution of validly published genera among the six ALMA soil samples. The double hierarchical dendrogram shows the actinobacterial distribution and the heatmap represents the relative percentage of each actinobacterial genus within each sample. The relative abundance values are indicated by the colour intensity as shown in the legend on the top left corner (JPEG 1435 kb) [file 792_2017_976_MOESM1_ESM.jpg]
